# “Sustainable Development: The Way for Future, Where are we?”

**DOI:** 10.4103/0970-0218.58381

**Published:** 2009-10

**Authors:** Rinku Sharma

**Affiliations:** Department of Community Medicine, Lady Hardinge Medical College, Delhi, India

Sustainable development is a common agenda for global concern, which everybody agrees upon, but bringing this global concern into public policies is a difficult task. The most accepted definition of sustainable development according to the Brundtland's report is, *“To meet the needs of present without compromising the ability of future generations to meet their own needs”. It advocated the idea of “sustainable growth”.*(1) According to *The World Conservation Strategy* report (1980), by the International Union for the Conservation of Nature and Natural Resources (IUCN), for development to be sustainable it must *take into account the social and economic factors as well as the ecological ones.*

India is presently emerging as an economic superpower, but in contrast, there is another profile of India. We constitute around 17% of the world's population, but account for about 35% of the poor and 40% of the illiterates in the world.(2) Experiences from the economic reform indicate that while there have been improvements in economic growth, foreign exchange, IT revolution, export growth, and so on, inequality in income distribution has been growing simultaneously (ratio of urban to rural income is 4.5).(2) Exclusion from benefits of economic revolution has been continued in terms of low agricultural growth (agriculture's share in GDP has been reduced to half, with no decrease in dependent population in the agricultural sector(2)), low quality employment growth, concentration of poverty in certain groups (SC / ST), occupation (agricultural and casual labor), and region; and inadequate development of women and children. Our sex ratio continues to remain favorable to men. Studies based on hospital statistics in South Delhi indicate that sex-ratio at birth is as low as 500 females per 1000 males,(3) All the above factors have resulted in the widening of economic and social disparity, which is a threat to sustainable development. The present economic growth helps to create more opportunities for the more educated section of the upper and middle class, with a ‘trickle-down’ effect on a section of the poor.

In India around 700 million people in the rural area are directly dependent on climate-sensitive sectors (agriculture, forests, and fisheries) and natural resources (such as water, biodiversity, mangroves, coastal zones, grasslands) for their subsistence and livelihoods. Climate change and its effects will further reduce the adaptive capacity of dry land farmers, forest dwellers, fisherfolk, and nomadic shepherds, which is already very low.(4) Water, soil, and air, which are the vital environmental sources for maintaining life have been shrinking alarmingly. Annual per capita availability of renewable freshwater has been decreasing from 5,277 m^3^ in 1955 to 1,820 m^3^ in 2001.(5) The main reasons for the water crisis are increasing demand, zonal disparity in distribution, lack of ethical framework for use, inadequate knowledge and resources, major land-use changes, long-term water level decline, and increase in salinity and pollution. India, with a large percentage of its land under agriculture, is also prone to the vagaries of weather conditions and climate change. About 228 Mha of its geographical area (nearly 69%) falls within the dry land (arid, semi-arid, and dry sub-humid) region and 142 Mha (68% of the total cultivated area) in the country is rain fed.(6)

To meet the challenging situation of widening economic and social disparity, inclusive growth is the best tool, but it is a dream without improvement in agricultural growth, employment generation, poverty reduction, and involvement of the social sector (health, education, and women empowerment). We must learn from China in this regard. Elements of the successful experience of the Chinese such as, high and labor-releasing agricultural growth, favorable income distribution through broad-based agricultural growth, availability of infrastructure, higher levels of literacy and skills, inducements for the location of enterprises in rural areas, and easy access to credit and inputs for the poor section of society, are extremely relevant for developing countries. Women empowerment through replacing the “Life-Cycle Approach” of the girl child, which has a prime objective of marriage and motherhood by a “Capability Approach” – as propagated by Amartya Sen, where the girl child's contributions both in economic and social terms are given due recognition. All Acts and Schemes related to the girl child, therefore, need to be thoroughly reviewed to raise the status of the girl child as an asset rather than burden, for example, conditional cash and non-cash transfer scheme, and so on.

Concerted and sustained efforts are required to meet the challenges resulting from climate change and its effects. Ground water conservation practices like construction of khadin (popular in Maharashtra, Andhra Pradesh, Madhya Pradesh, Tamil Nadu, Karnataka, and Gujarat), check dams, farm ponds, recharge shafts, injection wells (in coastal region and to combat problems of heavily pumped out aquifers), and contour trenching, to arrest surface run-off at elevations, and similarly surface water conservation techniques, like construction of ooranies (surface water collection ponds with improved catchments, commonly found in Tamil Nadu), are important measures to tackle problems of water scarcity and the decreasing ground water table. Generation of awareness and training among the masses for water conservation via roof top rain water harvesting and threshing floors can also be implemented. Involvement of the Gram Panchayat / Village Health and Sanitation Committee for operation, maintenance, and surveillance of water quality, as in the National Rural Drinking Water Quality Monitoring and Surveillance Project, can have a major impact. Other measures like recycling and reuse of water, using water-efficient household equipment such as low volume flushing cisterns, proper metering of water, rational tariff, and the concept of a water-efficient home, would reduce water demand and encourage conservation.

Integrated development of drought-prone areas can be done by long-term preventive measures like afforestation, pasture development, and livestock management, (by growing better top feed species, which can survive annual droughts and provide rich fodder). Contingency crop planning can be implemented by growing various combinations of crops, fruits, trees, and grasses, to minimize the risk of crop failure and to provide stability to farm income. Efficient land management and irrigation technologies like sprinklers and drip systems should be popularized, which aim at maximizing the production per unit of irrigation water. Other measures like human and livestock population management and generation of alternate ways of non-farm employment can go long way. A study titled, “Comprehensive Assessment of Watershed Programmes in India” by the International Crops Research Institute for the Semi-Arid Tropics (ICRISAT), Hyderabad, has identified the reduction of wastelands by about 8.58 Mha during 2000 and 2005, by using various techniques of integrated development of the drought prone area.(7) The *National Rural Employment Guarantee Act* (NREGA) is presently one of the most credible programs that deals with chronic poverty and improving sustainable development in rural areas. Under NREGA, up to two-third of the activities are for water conservation (52%) and land development (14%), a step toward sustainable development.(7)

For improving the present ecological conditions, India has taken a number of stringent steps, such as, (1) registration of the largest number of Clean Development Mechanism (CDM) projects (31%) in the world.(8) CDM or carbon credits is a mechanism devised under the Kyoto Protocol to award encashable points to eco-friendly projects, on the basis of carbon emissions they control. (2) A Mumbai start-up, Sustainable Technologies and Environmental Projects Ltd (STEPS), has discovered a way to convert plastic, organic, and electronic waste into petroleum without the usual harmful residue. Such plants, which cost US$ 2 - 3 million each, can produce up to 25,000 liters of petroleum a day, at an operating cost of Rs. 12 per liter (excluding cost of raw materials).(8) India is also trying to replace 10% of its transport fuels with environment friendly biofuels (mixing ethanol, doping diesel, and nonedible oil) in the next 10 years, to cut carbon emissions.(9) (3) A new initiative of the US Green Building Council-Leadership in Energy and Environmental Design (USGBC-LEED) – an organization that uses the 69-point criteria to award certificate at the platinum, gold, and other levels to buildings. Today, our country has over 25 million square feet of registered green building expanse, which is all set to touch 100 million square feet by 2010-12. ITC Green Center in Gurgaon, is the world's largest green building, with a space of 170,000 square feet, and the first non-commercial complex in our country to be awarded a platinum rating by USGBC-LEED.(4) (4) People participation in the form of Corporate Social Responsibility (CSR), as a means of reducing the social and economic disparity and improving ecological conditions through various activities via the corporate sector like health, education, natural resource management, infrastructure development, community support, non-farm and farm-based livelihood development.

We have a path for sustainable development, but unless all our methods are directed toward it, we cannot achieve sustainable development.
